# Supplementary material for: Rapid turnover of life-cycle-related genes in the brown algae
Source: Genome Biol. 2019 Feb 14;20:35. doi: 10.1186/s13059-019-1630-6 (PMC6374913; doi:10.1186/s13059-019-1630-6)
Supplement: Supplementary file 3 — Protein alignments, corresponding Gblocks html file and CDS sequences for the lineage conserved gametophyte-biased genes. (ZIP 2109 kb) [file 13059_2019_1630_MOESM3_ESM.zip › Con_GA_alignments/Ec-06_003350.aa_ali.fasta-gb.htm]

Ec-06\_003350.aa.fasta.trans.aa\_ali.fasta


## Gblocks 0.91b Results

Processed file: **Ec-06\_003350.aa.fasta.trans.aa\_ali.fasta**  
Number of sequences: **2**  
Alignment assumed to be: **Protein**  
New number of positions: **153** (selected positions are underlined in blue)

```
                         10        20        30        40        50        60
                 =========+=========+=========+=========+=========+=========+
Ec-06_003350     MMAKMANVALVALTLAAGGLAEESRSLASGVWGAWKSERVKSKYFSELLADGDTVELLTH
MSTRG.48581.1    -----------------------------------------SMYFSELLENDETVELLTH
                                                          ###################


                         70        80        90       100       110       120
                 =========+=========+=========+=========+=========+=========+
Ec-06_003350     GPFTISAACGTLGGEAEVRLLLTVTADSDADEWVYGYPDFLPQDYYNSCVGTTAAGTVSD
MSTRG.48581.1    GPFTISAGCGVIEDQPRVRLFMTVTADSDADEWVYGFPDFLPLDYYNSCLGTTPAGTVSD
                 ############################################################


                        130       140       150       160       170       180
                 =========+=========+=========+=========+=========+=========+
Ec-06_003350     TCVVWGTVSGSGFPDDAGAYTSLSGYYVSFGGPSTIGFNKEALSNVQYLTYANGFTAEDV
MSTRG.48581.1    TCIVWSTVSGEGS--HPGAFTSLSGYYIGLGGSSTIGFNKEYLMN--DATYQNAYSADDI
                 ############     ############################    ###########


                        190       200
                 =========+=========+===
Ec-06_003350     AAFPNDCAISGELTYNRASGDDP
MSTRG.48581.1    AAFPTDCAISGELTWNRASGDDP
                 #######################
```

```
Parameters used
Minimum Number Of Sequences For A Conserved Position: 2
Minimum Number Of Sequences For A Flanking Position: 2
Maximum Number Of Contiguous Nonconserved Positions: 8
Minimum Length Of A Block: 10
Allowed Gap Positions: None
Use Similarity Matrices: Yes
```

```
Flank positions of the 3 selected block(s)
Flanks: [42  132]  [138  165]  [170  203]  

New number of positions in Ec-06_003350.aa.fasta.trans.aa_ali.fasta-gb:  153  (75% of the original 203 positions)
```
